# Molecular analysis of vector-borne pathogens in Eurasian badgers (*Meles meles*) from continental Europe

**DOI:** 10.1186/s13071-024-06515-y

**Published:** 2024-11-04

**Authors:** Zoë Tess Lara Lindhorst, Sebastian Brandstetter, Maria Sophia Unterköfler, Barbara Eigner, Joachim Spergser, Marc Colyn, Peter Steinbach, Duško Ćirović, Nikica Šprem, Tomislav Dumić, Vincenzo Veneziano, Franz Müller, Josef Harl, Georgiana Deak, Angela Monica Ionică, Mike Heddergott, Hans-Peter Fuehrer

**Affiliations:** 1 Institute of Parasitology, Department of Biological Sciences and Pathobiology, University of Veterinary Medicine Vienna, Vienna, Austria; 2 Institute of Microbiology, Department of Biological Sciences and Pathobiology, University of Veterinary Medicine Vienna, Vienna, Austria; 3 UMR 6553 Ecobio, Station Biologique, CNRS–Université de Rennes 1, Paimpont, France; 4 Musée National d’Histoire Naturelle, Luxembourg, Luxembourg; 5 Faculty of Chemistry, Georg-August University of Göttingen, Göttingen, Germany; 6 Faculty of Biology, University of Belgrade, Belgrade, Serbia; 7 Department of Fisheries, Apiculture, Wildlife Management and Special Zoology, Faculty of Agriculture, University of Zagreb, Zagreb, Croatia; 8 Department of Wildlife Management and Nature Conservation, Karlovac University of Applied Sciences, Karlovac, Croatia; 9 Department of Veterinary Medicine and Animal Productions, University of Naples Federico II, Naples, Italy; 10 Wildlife Biology Working Group, Justus-Liebig-Universität Gießen, Giessen, Germany; 11 Institute of Pathology, Department of Biological Sciences and Pathobiology, University of Veterinary Medicine Vienna, Vienna, Austria; 12 Department of Experimental Pathology and Laboratory Animal Pathology, Medical University Vienna, Vienna, Austria; 13 Department of Parasitology and Parasitic Diseases, University of Agricultural Sciences and Veterinary Medicine of Cluj-Napoca, Cluj-Napoca, Romania; 14 Clinical Hospital of Infectious Diseases of Cluj-Napoca, Cluj-Napoca, Romania

**Keywords:** Vector-borne pathogens, Badger, *Babesia*, *Trypanosoma*, *Mycoplasma*, *Ehrlichia*

## Abstract

**Background:**

Vector-borne pathogens (VBPs) are increasing in significance in veterinary medicine and public health settings, with wildlife playing a potentially crucial role in their transmission. Eurasian badgers (*Meles meles*) are widely distributed across Europe. However, information currently available on the prevalence of VBPs in badgers is limited. The objective of the current study was to investigate the occurrence of Anaplasmataceae, *Bartonella* spp., *Mycoplasma* spp., *Rickettsia* spp., Piroplasmida, Trypanosomatida and Filarioidea in badgers and subsequently, based on the results, assess the potential risk to domestic animals, other wildlife and humans.

**Methods:**

Between 2017 and 2021, blood or spleen samples from 220 badgers were collected in nine continental European countries: Austria (*n* = 7), Bosnia and Herzegovina (*n* = 2), Croatia (*n* = 22), France (*n* = 44), Germany (*n* = 16), Hungary (*n* = 7), Italy (*n* = 16), Romania (*n* = 80) and Serbia (*n* = 26). VBPs were identified by performing PCR analysis on the samples, followed by Sanger sequencing. Additionally, to distinguish between different *Babesia* lineages we performed restriction fragment length polymorphism (RFLP) analysis on piroplasm-positive samples, using HinfI as restriction enzyme. A phylogenetic analysis was performed on *Mycoplasma* spp.

**Results:**

The pathogens identified were *Babesia* sp. badger type A (54%), B (23%), and C (37%); *Trypanosoma pestanai* (56%); *Mycoplasma* sp. (34%); Candidatus Mycoplasma haematomelis (8%); *Candidatus* Mycoplasma haematominutum (0.5%); and *Ehrlichia* spp. (2%). *Rickettsia* spp., *Bartonella* spp. and filarioid nematodes were not detected among the tested samples.

**Conclusions:**

The large sample size and diverse study populations in this study provide valuable insights into the distribution and epidemiology of the analyzed pathogens. Some of the VBPs identified in our study show high similarity to those found in domestic animals, such as dogs. This finding suggests that badgers, as potential reservoirs for these pathogens, may pose a threat not only to other wildlife but also to domestic animals in close vicinity. Continuous surveillance is essential to monitor VBPs in wildlife as a means to enable the assessment of their impact on other wildlife species, domestic animals and human health.

**Graphical Abstract:**

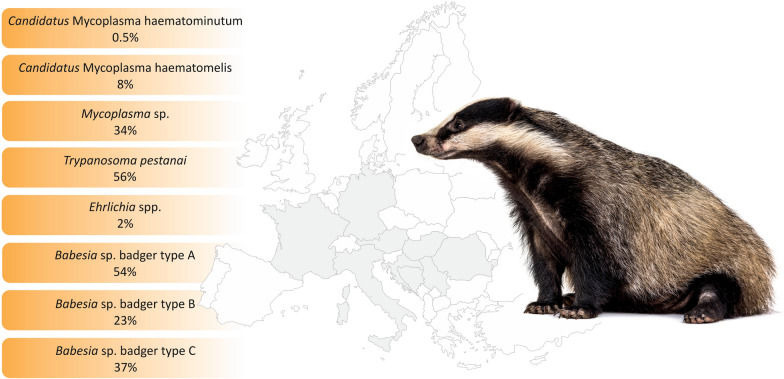

**Supplementary Information:**

The online version contains supplementary material available at 10.1186/s13071-024-06515-y.

## Background

Emerging infectious diseases have been increasingly recognized and deemed significant in Europe in recent years [1]. The primary reasons for the increasing emergence of vector-borne pathogens (VBPs) in European countries, which were previously not present or less prevalent, include globalization, urbanization, global trade, increased travel of humans and domestic animals and climatic changes [2, 3]. These substantial changes have led to the widespread expansion of VBPs and arthropods acting as their vectors [4]. Wildlife serve as potential reservoirs and may therefore play a crucial role in the transmission of these pathogens. Due to the zoonotic potential of many VBPs and their capability to infect domestic animals, VBPs can be highly important factors in both veterinary and human medicine [5].

The Eurasian badger (*Meles meles*) is among the most prevalent medium-sized carnivores throughout Europe [6], where it mainly inhabits woodlands [7, 8]. Despite their typically shy and elusive behavior towards humans, badgers, along with other wildlife, are regularly found near human settlements, influenced by factors such as habitat loss and food availability [7, 9]. To date, limited information is available on the prevalence of VBPs in badgers, particularly in populations beyond the UK [10]. However, the close coexistence of humans and badgers, with the latter possibly serving as reservoirs for numerous pathogens [11–14], could pose a significant threat to the health of both humans and domestic animals. For example, badgers are considered to be an important source of bovine tuberculosis in the UK due to the possible transmission of *Mycoplasma bovis* to cattle [15].

Among the most important parasitic pathogens found in badgers are members of the order Piroplasmida. For example, badger-associated *Babesia* parasites, which are suspected to belong to the *Babesia microti* group, have been detected in several European countries. *Babesia* sp. isolates badger type A, type B [1, 16–25] and *Babesia* sp. badger, which was labeled type C in one study [25], have been identified. In addition to badgers, badger-associated *Babesia* have also been described in other carnivores in Europe [16, 19, 26]. Ixodid ticks act as the main vectors of *Babesia* spp. [27]. Another parasite detected in badgers is *Trypanosoma* (*Megatrypanum*) *pestanai*, a species that was identified in badgers from the UK, France and Italy [28–30], and in a dog (*Canis lupus familiaris*) from Germany [31]. The role of badger fleas (*Paraceras melis*) as vectors for *T. pestanai* had been confirmed [32] when *T. pestanai* was also detected in ixodid ticks for the first time in Italy [29]. In recent years, Eurasian badgers have been confirmed as new hosts for the filarioid nematodes *Dirofilaria immitis* [33] and *Dirofilaria repens* [11, 34]. However, the role of the badger as a definite host and a reservoir for *D. immitis* and *D. repens* remains unclear and requires further investigation. The most important bacterial VBPs in European carnivores include *Mycoplasma* spp., Anaplasmataceae, *Borrelia* spp., *Bartonella* spp. and *Rickettsia* spp. [5, 35, 36]. Hemotropic mycoplasmas (hemoplasmas) have been described in Eurasian badgers [37] and a Japanese badger (*Meles meles anakuma*) [38]. Infections with *Anaplasma phagocytophilum*, *Ehrlichia* sp. [1, 39, 40] and *Candidatus* Neoehrlichia sp. [18, 41] have been identified in badgers in different European countries including, for example, Italy and the Netherlands. Other pathogens that were detected in badgers are *Bartonella* spp. [42], *Rickettsia* spp. [43], and *Borrelia* spp. [13, 44].

The objective of our research was to investigate the occurrence of Anaplasmatacea, *Bartonella* spp., *Mycoplasma* spp., *Rickettsia* spp., Piroplasmida, Trypanosomatida and Filarioidea in badgers across nine European countries. The findings of this study provide valuable insights into the epidemiology of these pathogens in badger populations, thereby aiding in the assessment of potential cross-species transmission risks to domestic animals, wildlife and humans.

## Methods

### Sample collection

**Fig. 1:**
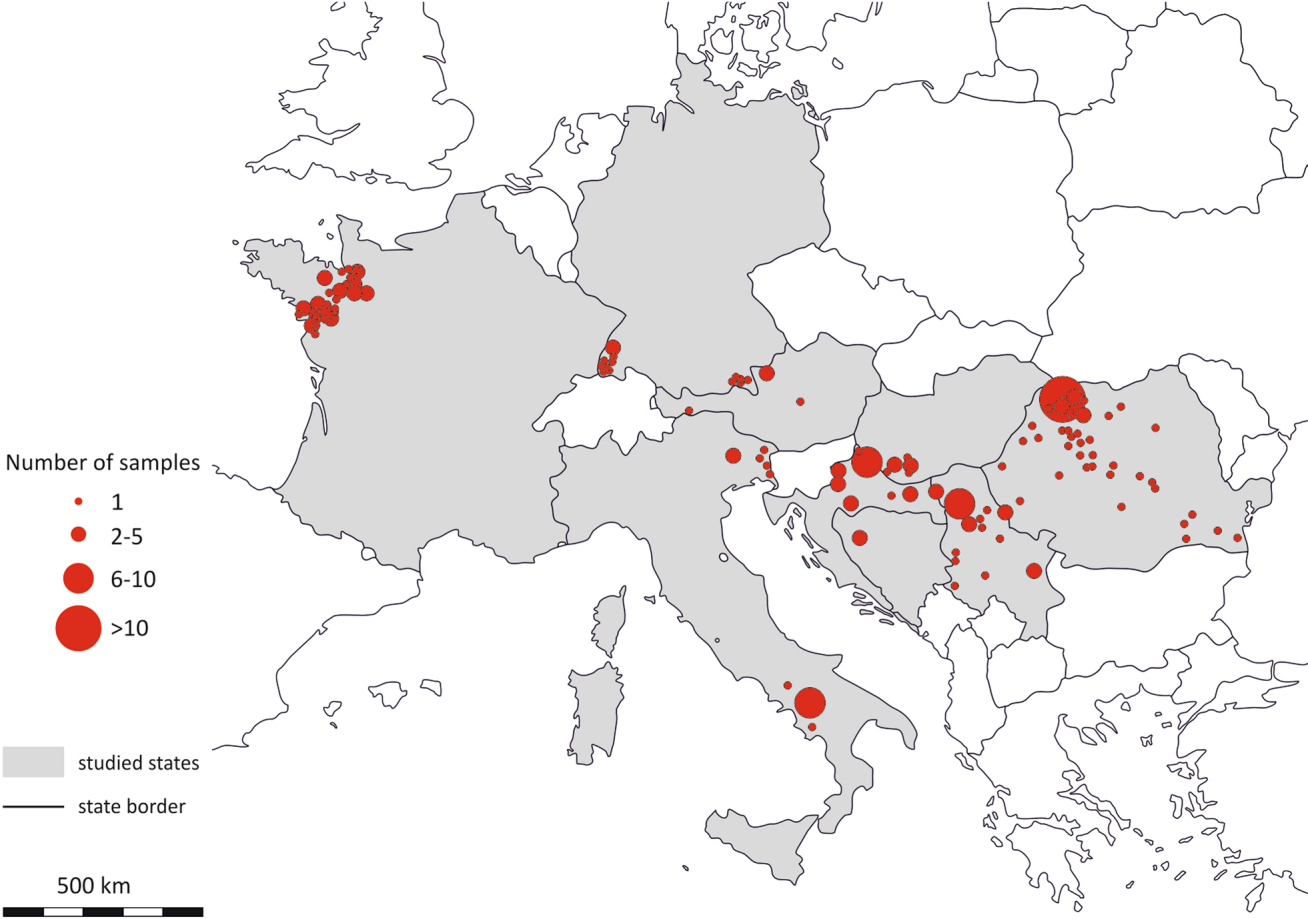
Geographical distribution of the Eurasian badgers (*Meles meles*) examined in this study (*n* = 220). The size of the circles indicates the number of samples per locality

Blood (*n* = 81) or spleen (*n* = 139) samples from 220 Eurasian badgers were analyzed for the presence of VBPs. No animal was killed for the study; all samples were collected from individuals that were found dead and were taken from fresh cadavers (mainly between 24 and 48 h after death). The samples were collected from July 2017 to August 2021 in nine different European countries: Austria (*n* = 7), Bosnia and Herzegovina (*n* = 2), Croatia (*n* = 22), France (*n* = 44), Germany (*n* = 16), Hungary (*n* = 7), Italy (*n* = 16), Romania (*n* = 80) and Serbia (*n* = 26) (Fig. 1). All samples were kept frozen at -20 °C in 70% ethanol until the analysis at the University of Veterinary Medicine, Vienna. During necropsy, all individuals were examined to determine their sex and age, based on dentition stage and tooth wear [45]. According to the age classification, the animals were then divided into two groups: juvenile (aged ≤ 12 months) and adult (aged > 12 months).

### DNA extraction, PCR amplification and sequencing

DNA was extracted from the samples using the DNeasy Blood & Tissue kit (250) (QIAGEN, Hilden, Germany), following the manufacturer’s instructions. Pathogen detection was carried out following seven established broad-range PCR assay protocols (Table 1; Additional file 1: Table S1), with each protocol including positive and negative controls. The PCR assays targeted sections of the *16S* ribosomal RNA (rRNA) in *Mycoplasma* spp. and Anaplasmataceae, the *18S* rRNA in Piroplasmida and Trypanosomatida, the *16S*-*23S* rRNA in *Bartonella* spp., the *23S*-*5S* rRNA intergenic spacer in *Rickettsia* spp. and the *COI* gene in Filarioidea [46–53]. PCRs were run using GoTaq™ DNA Polymerase (Promega, Madison, WI, USA).

**Table 1:** Oligonucleotide sequences of primers used in the present study

Target organism (genetic marker)	Primer sequences (5′ → 3′)	Product size (bp	Annealing temperature	Reference
*Mycoplasma* spp. (*16S* rRNA)	HBT-F: ATACGGCCCATATTCCTACG HBT-R: TGCTCCACCACTTGTTCA	600	60 °C	[46]
*Mycoplasma* spp. (*16S* rRNA)	UNI_16 S_mycF: GGCCCATATTCCTACGGGAAGCAGCAGT UNI_16 S_mycR: TAGTTTGACGGGGGGTGTACAAGACCTG	1000	56 °C	[53]
Piroplasmida (*18S* rRNA)	BTF1: GGCTCATTACAACAGTTATAG BTR1: CCCAAAGACTTTGATTTCTCTC	930	52 °C	[48]
	BTF2: CCGTGCTAATTGTAGGGCTAATAC BTR2: GGACTACGACGGTATCTGATCG	800	62 °C	
Trypanosomatida (*18S* rRNA)	Tryp_18S_F1: GTGGACTGCCATGGCGTTGA Tryp_18S_R1: CAGCTTGGATCTCGTCCGTTGA	~ 1320	56 °C	[51]
	Tryp_18S_F2: CGATGAGGCAGCGAAAAGAAATAGAG Tryp_18S_R2: GACTGTAACCTCAAAGCTTTCGCG	960	56 °C	
*Bartonella* spp. (*16S*-*23S* rRNA)	Bartgd_for: GATGATGATCCCAAGCCTTC B1623_rev: AACCAACTGAGCTACAAGCC	179	60 °C	[49]
*Rickettsia* spp. (*23S*-*5S* rRNA)	Ricketts_ITS_for: GATAGGTCGGGTGTGGAAG Ricketts_ITS_rev: TCGGGATGGGATCGTGTG	~ 400	52 °C	[52]
Anaplasmataceae (*16S* rRNA)	EHR16SD_for: GGTACCYACAGAAGAAGTCC EHR16SR_rev: TAGCACTCATCGTTTACAGC	345	54 °C	[50]
Filarioidea (*COI* gene)	COIint-F: TGATTGGTGGTTTTGGTAA COIint-R: ATAAGTACGAGTATCAATATC	668	52.3 °C	[47]

*COI* Cytochrome* c* oxidase I,* rRNA* ribosomal RNA

The PCR products were subjected to electrophoresis in 1.8% agarose gels with 4.2 µl Midori Green Advance DNA Stain (NIPPON Genetics EUROPE GmbH, Düren, Germany). PCR products of positive samples were sent to LGC Genomics (Berlin, Germany) for sequencing. The chromatograms were visually inspected and edited (using Chromas and GeneDoc) and compared against data from the NCBI GenBank and the Barcode of Life (BOLD; https://www.boldsystems.org/) databases.

In the case of Piroplasmida, a restriction fragment length polymorphism method (RFLP), using a HinfI restriction enzyme (Promega), was conducted on positive samples to distinguish between *Babesia* lineages and to detect mixed infections [48]. Three representative samples (one from each *Babesia* haplotype identified through nested PCR) were selected for sequencing, and these were then used as references for the remaining samples.

### Phylogenetic analysis

One *Mycoplasma* sequence obtained in the present study (GenBank accession no.: OQ749679) was used to search for similar sequences using the BLAST function on NCBI GenBank, setting the number of maximum target sequences to 5000. The filter was set to 82–100% identity and 98–100% query coverage. The sequences were aligned and sorted using the default option (FFT–NS–2) in MAFFT v.7.520 [54]. All sequences featuring obvious sequencing errors and ambiguous characters were removed from the alignment and excluded from the analysis.

To provide an overview of the diversity of haplotypes, maximum likelihood (ML) and Bayesian inference (BI) trees were calculated based on an alignment containing 392 sequences (1030 nucleotide positions). Gaps were removed from the alignment using TrimAl v.1.3 (http://phylemon2.bioinfo.cipf.es/; [55]), and sequences were collapsed to haplotypes using DAMBE v.7.3.32, leaving 237 haplotypes (963 nucleotide positions). The tree was rooted with a sequence of *Malacoplasma iowae* (GenBank accession no.: CP129195).

An ML bootstrap consensus tree (1000 replicates) was calculated using the W-IQ-TREE web server (http://iqtree.cibiv.univie.ac.at/; [56]) applying the model GTR + F + I + G4, which was suggested as the best fit for the data set in the model test according to the Bayesian inference criterion (BIC). The BI trees were calculated using MrBayes v.3.2.7 [57], applying the model GTR + G + I. The analysis was run for 10^6^ generations (2 runs each with 4 chains), sampling every thousandth tree. The first 25% of trees were discarded as burn-in and a 50% majority-rule consensus tree was calculated based on the remaining 750 trees. The ML and BI trees were jointly created using the BI tree as a template, and then graphically prepared indicating country and host information in CorelDRAW 2024 (Corel, Ottawa, ON, Canada).

### Statistical analysis

Statistical analysis was conducted using R version 4.3.2 ® Foundation for Statistical Computing, Vienna, Austria). A Pearson's Chi-squared (*χ*^2^) test was conducted to assess the correlation between the detection of pathogens and the sex and age of the animals. The association between pathogen detection and country of origin, and month and year of sample collection, respectively, was assessed using Fisher’s exact test. Effects were considered statistically significant if *P* < 0.05.

## Results

Pathogens detected in the 220 tested blood/spleen samples from badgers were Piroplasmida (195/220, 88.6%; 95% confidence interval [CI] 0.844–0.928), Trypanosomatida (123/220, 55.9%; 95% CI 0.493–0.625), *Mycoplasma* spp. (107/220, 48.6%; 95% CI 0.42–0.552) and Anaplasmataceae (4/220, 1.8%; 95% CI 0.001–0.036). *Dirofilaria* spp. and other filarioid helminths, *Rickettsia* spp. and *Bartonella* spp. were not detected in the tested samples. No significant correlation was found between pathogen occurrence and age (*P* = 0.219; odds ratio [OR] Infinite, 95% CI 0.533-Infinity), sex (*χ*^2^ = 0.008, *df* = 1, *P* = 0.928), year of sample collection (*P* = 0.439) and month of sample collection (*P* = 0.817). There was a significant correlation between pathogen occurrence and country of origin (*P* = 0.013). Additional statistical analyses are presented in Additional file 1: Table S2.

**Fig. 2:**
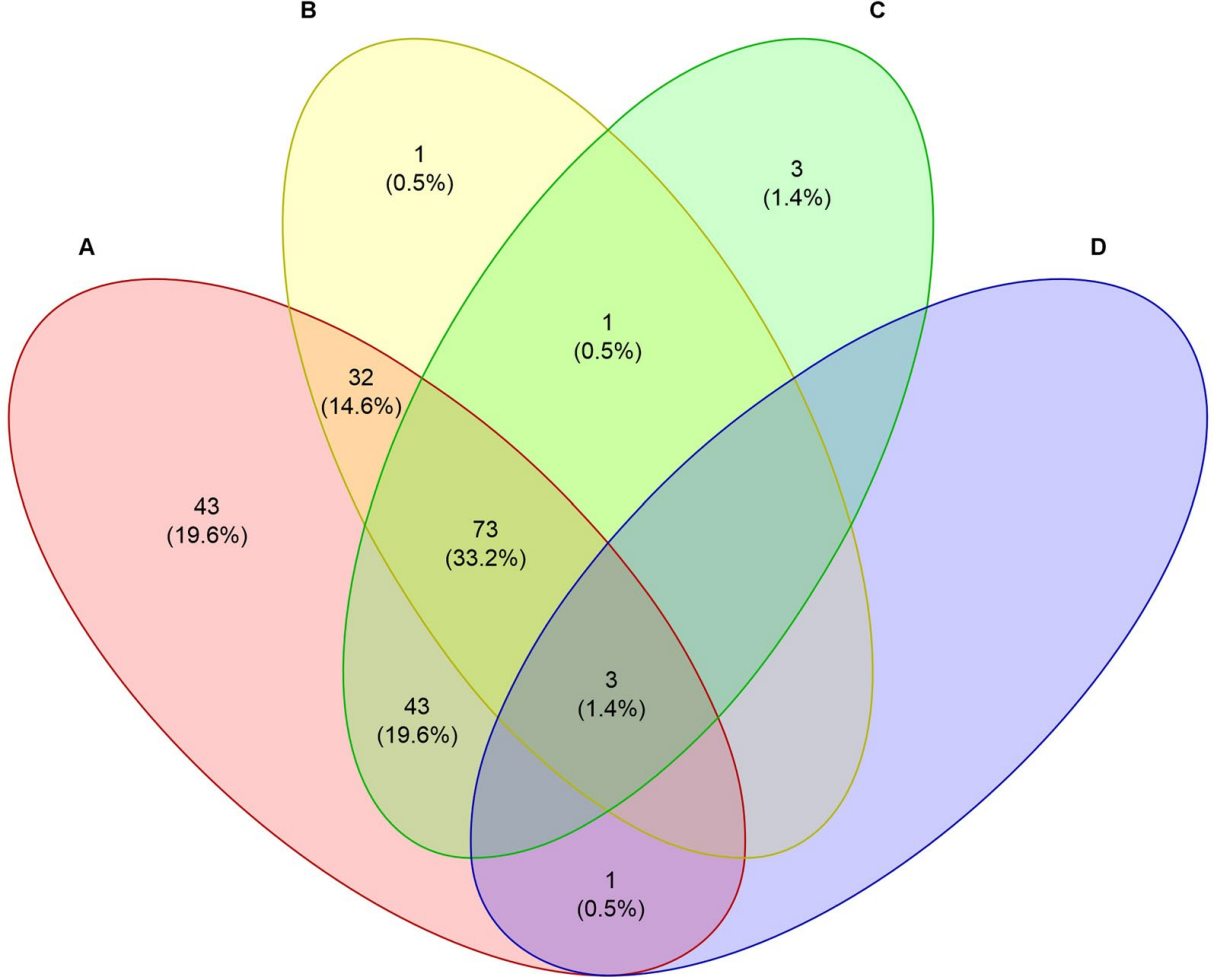
Co-infection scheme of detected pathogens. Numbers represent counts of Eurasian badgers (*Meles meles*) with respective pathogens detected. Percentages represent the proportion of positive badgers among all badgers tested (*n* = 220). **A**, *Babesia* sp.; **B**, hemotropic mycoplasmas; **C**, *Trypanosoma pestanai*; **D**, *Ehrlichia* sp.

**Fig. 3:**
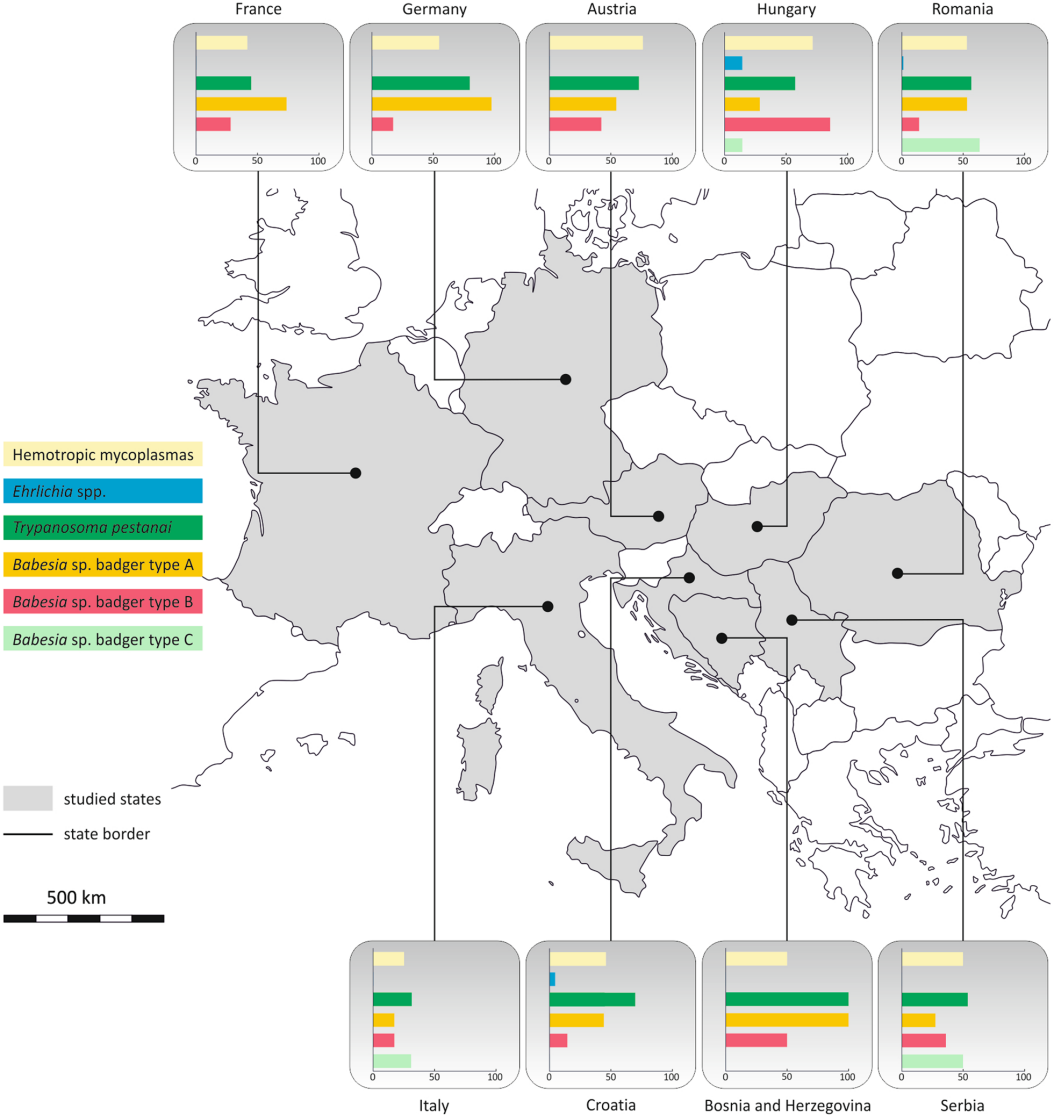
Map of the Eurasian badger (*Meles meles*) samples used for this study, showing the prevalence by state in Europe. The bar charts show the prevalence (in %) of hemotropic mycoplasmas, *Ehrlichia* sp., *Trypanosoma pestanai*, *Babesia* sp. badger type A, *Babesia* sp. badger type B and *Babesia* sp. badger type C by study state

Of the 220 badger samples tested, 47 (21.4%; 95% CI 0.16–0.268) tested positive for one pathogen only, and 77 (35.0%; 95% CI 0.287–0.413) and 76 (34.6%; 95% CI 0.287–0.413) showed a co-infection with two and three pathogens, respectively (Fig. 2). The highest prevalence of pathogens was found in Austria, Bosnia and Herzegovina, Croatia, Germany and Hungary (each 100%), followed by Romania (94%), Serbia (92%), France (84%) and Italy (63%) (Fig. 3).

**Fig. 4:**
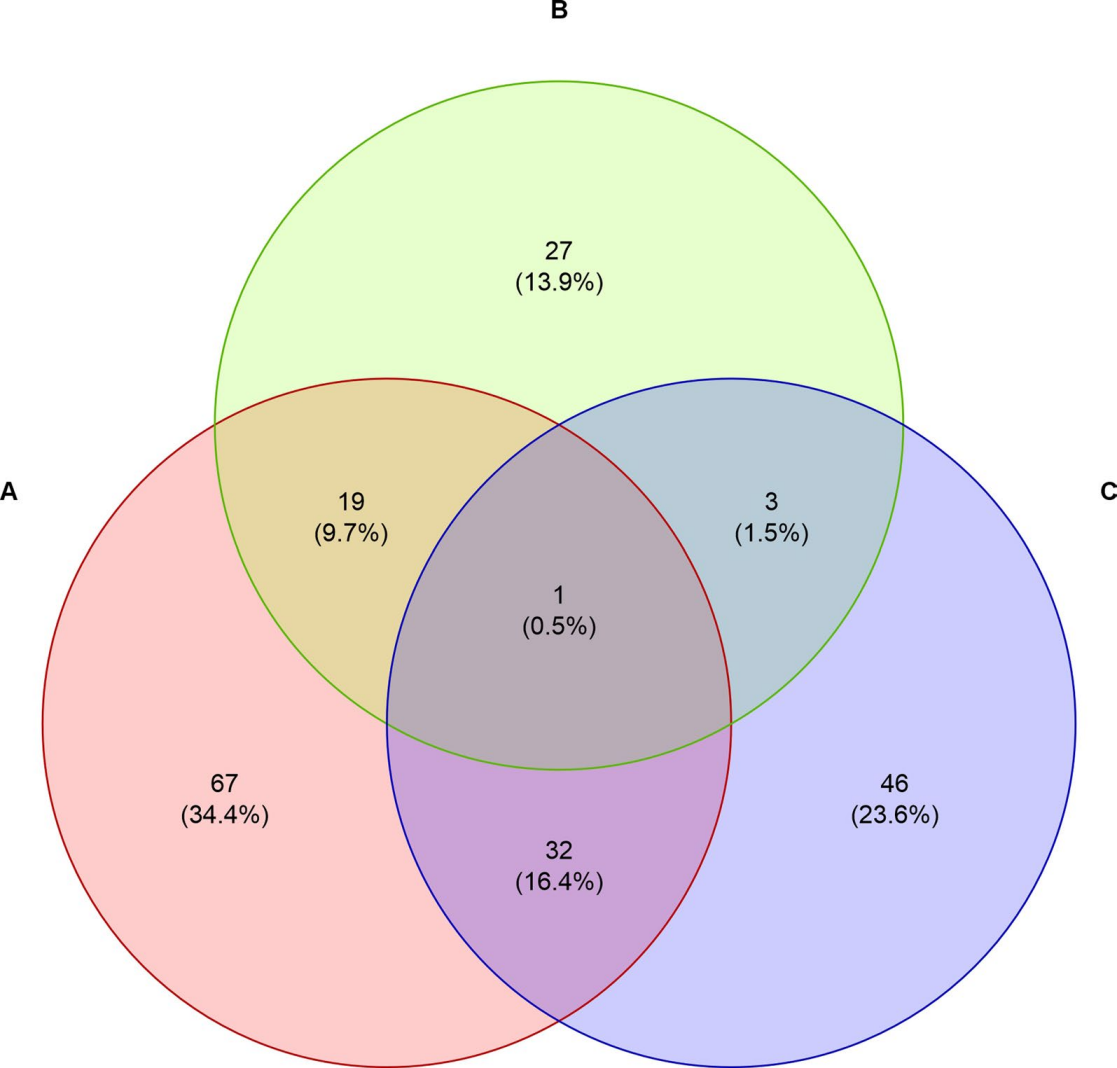
Co-infection scheme of detected *Babesia* sp. lineages. Numbers represent counts of Eurasian badgers (*Meles meles*) with the respective haplotype(s) detected. Percentages represent the proportion of positive badgers among Piroplasmida-positive badgers (*N* = 195). **A**, *Babesia* sp. badger type A; **B**, *Babesia* sp. badger type B; **C**, *Babesia* sp. badger type C

Among the 195 samples positive for Piroplasmida, 119 (61.0%; 95% CI 0.542–0.679) could be assigned to *Babesia* sp. badger type A (GenBank accession number: KT223484), 50 (25.5%; 95% CI 0.12–0.318) to *Babesia* sp. badger type B (GenBank accession number: KT223485) and 82 (42.1%; 95% CI 0.351–0.49) to *Babesia* sp. badger type C (GenBank accession number: MG799847). Of these 195 Piroplasmida-positive badgers, 140 (71.8%; 95% CI 0.655–0.781) showed an infection with one lineage only, 54 (27.7%; 95% CI 0.214–0.34) showed an infection with two lineages and one (0.5%; 95% CI 0–0.015) showed an infection with all three lineages simultaneously (Fig. 4). Three sequences, representative for the three haplotypes found in the present study, were uploaded to NCBI GenBank (accession nos.: PP621229–PP621231) (Table 2). The representative samples for type A and type B showed 100% identity to *Babesia* sp. badger type A and *Babesia* sp. badger type B, respectively, found in badgers from Spain. The representative sample for type C showed 100% identity to *Babesia* sp. badger “isolate Badger-1,” found in a badger from China (Table 2). The prevalence of badger-associated *Babesia* spp. was 100% in Bosnia and Herzegovina, Germany and Hungary, followed by Serbia (92%), Romania (90%), France (84%), Austria (71%) and Italy (63%) (Fig. 3).

**Table 2:** Sequencing results for *Ehrlichia* spp., *Trypanosoma pestanai* and Eurasian badger-associated *Babesia* spp. and their closest relationship

Accession no. (this study)	Haplotype (this study)	Country (this study)	Reference haplotype	Reference host	Reference country	Reference accession no.	Identity (in %)
PP595801	Uncultured *Ehrlichia* sp.	Croatia	Uncultured *Ehrlichia* sp.	*Canis lupus familiaris*	Hungary	MH020203	100.0
PP595227	*Trypanosoma pestanai*	Austria	*Trypanosoma pestanai*	*Meles meles*	France	AJ009159	100.0
PP595228	*Trypanosoma pestanai*	Austria	*Trypanosoma pestanai*	*Meles meles*	France	AJ009159	100.0
PP595229	*Trypanosoma pestanai*	Romania	*Trypanosoma pestanai*	*Meles meles*	Italy	MZ144610	98.7
PP621229	*Babesia* sp. badger type A	France	*Babesia* sp. badger type A	*Meles meles*	Spain	KT223484	100.0
PP621230	*Babesia* sp. badger type B	Germany	*Babesia* sp. badger type B	*Meles meles*	Spain	KT223485	100.0
PP621231	*Babesia* sp. badger type C	Croatia	*Babesia* sp. badger	*Meles meles*	China	MG799847	100.0

All 118 samples that tested positive for Trypanosomatida were infected with *T. pestanai*. Of these, 82 (69.5%; 95% CI 0.612–0.778) of the sequences were 99.1–100% identical to those of *T. pestanai* from a badger in France (GenBank accession number: AJ009159), and 22 (18.6%; 95% CI 0.116–0.257) were 98.2–100% identical to *T. pestanai* detected in a badger in Italy (GenBank accession number: MZ144610). Three representative sequences were uploaded to NCBI GenBank under the accession numbers PP595227–PP595229 (Table 2). The sequence quality of the remaining 14 samples was poor and therefore could not definitely be assigned to a species, although the sequences were similar to those of *T. pestanai*. The prevalence of *T. pestanai* was highest in Bosnia and Herzegovina (100%), followed by Germany (75%), Austria (71%), Croatia (68%), Romania (58%), Hungary (57%), Serbia (54%), France (45%) and Italy (31%) (Fig. 3).

The sequences of three samples that tested positive for Anaplasmataceae were 100% identical and one was 99.5% identical to *Ehrlichia* sp. found in a dog from Hungary (GenBank accession number: MH020203). Due to the low sequence qualities of two sequences, only one was uploaded to GenBank under the accession number PP595801 (Table 2). *Ehrlichia* sp. was found in Croatia, Hungary and Romania (*n* = 2, 1 and 1, respectively) (Fig. 3).

**Fig. 5:**
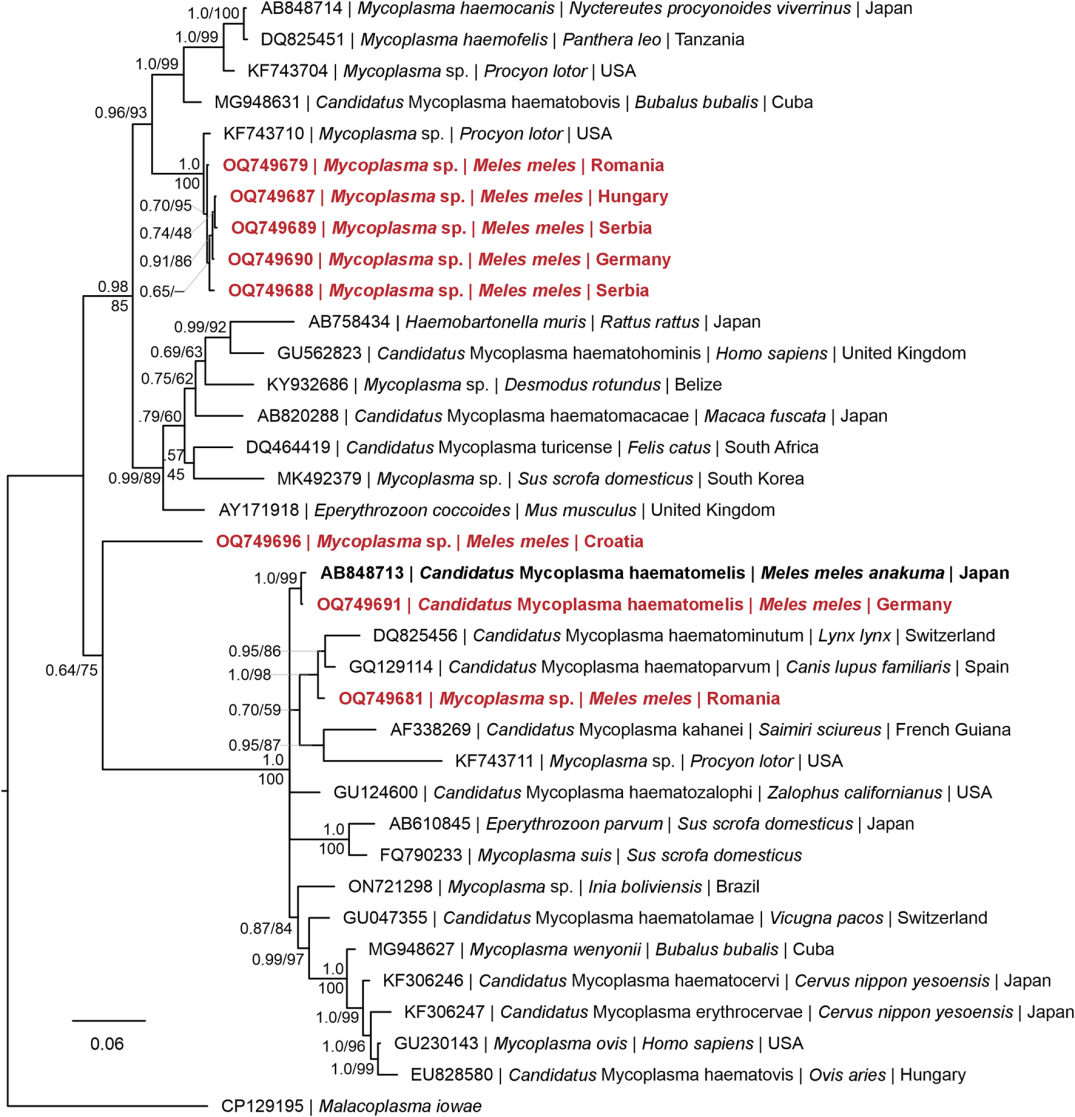
Bayesian inference tree featuring *16S* rRNA sequences (963 nucleotide positions) of selected *Mycoplasma* spp. Nodes are marked with Bayesian posterior probabilities and maximum likelihood bootstrap values. Accession numbers, species name, host and country are provided for every sequence. Sequences which are written in bold are from *Meles meles*, and sequences marked in red were obtained in this study. The scale bar indicates the expected mean number of substitutions per site according to the model of sequence evolution applied. For reasons of clarity, the number of sequences has been reduced to up to 4 sequences per clade. The full phylogenetic tree can be found in Additional file 2: Figure S1

Of the samples that tested positive for *Mycoplasma* spp., 19 were subjected to additional PCRs (each with different primers amplifying larger PCR products), and the resulting sequences were used for the phylogenetic analysis. Due to the low quality of the sequences, two were excluded from the analysis. The remaining 17 sequences were uploaded to NCBI GenBank under the accession numbers OQ749679–OQ749684 and OQ749687–OQ749697) (Table 3) and used for phylogenetic analysis (Fig. 5; Additional file 2: Figure S1). Among the 107 samples that tested positive for *Mycoplasma* spp. during the primary PCR, only two sequences showed 100% identity to sequences uploaded to GenBank, one (GenBank accession number: OQ749681) to *Mycoplasma* sp. from a European wild cat (*Felis silvestris silvestris*) in Bosnia and Herzegovina (GenBank accession number: MF614158), and one (GenBank accession number:OQ749683) to *Candidatus* Mycoplasma haematomelis found in a domestic cat (*Felis catus*) from Italy (GenBank accession number: KR9055451). However, the results from both samples differed from those obtained in the secondary PCR, where they showed 98.8% identity to *Mycoplasma* sp. found in an American mink (*Neogale vison*) from Chile (GenBank accession number: MT462252), and 99.6% to *Mycoplasma* sp. found in a raccoon (*Procyon lotor*) from the USA (GenBank accession number: KF743733), respectively. The remaining 105 samples showed identities of 96.04–99.8% (Table 4) and notable differences from those observed by the second PCR. The prevalence of *Mycoplasma* spp. was highest in Hungary (71%), followed by Austria (57%), Germany (56%), Romania (53%), Bosnia and Herzegovina (50%), Serbia (50%), Croatia (45%), France (43%) and Italy (25%) (Fig. 3).

**Table 3:** Sequencing results for *Mycoplasma* spp., using UNI_16 S_mycF and UNI_16 S_mycR primers, and their closest relationship, according to GenBank BLAST results

Number of samples	Accession number (this study)	Country (this study)	Reference haplotype	Reference host	Reference country	Reference accession number	Identity (in %)
5	OQ749691	Germany, France	*Candidatus* Mycoplasma haematomelis	*Meles meles anakuma*	Japan	AB848713	99.7
1	OQ749696	Croatia	*Mycoplasma* sp.	*Canis lupus familiaris*	Cambodia	ON620261	99.4
1	OQ749681	Romania	*Mycoplasma* sp.	*Neovison vison*	Chile	MT462252	98.8
5	OQ749679	Romania	*Mycoplasma* sp.	*Procyon lotor*	USA	KF743733	99.6
2	OQ749689	Serbia, Croatia	*Mycoplasma* sp.	*Procyon lotor*	USA	KF743733	99.5
1	OQ749690	Germany	*Mycoplasma* sp.	*Procyon lotor*	USA	KF743733	99.4
1	OQ749687	Hungary	*Mycoplasma* sp.	*Procyon lotor*	USA	KF743733	99.4
1	OQ749688	Serbia	*Mycoplasma* sp.	*Procyon lotor*	USA	KF743733	99.3

Of the samples that tested positive for *Mycoplasma* spp., 19 were subjected to additional PCRs, of which 2 sequences of minor quality were excluded from the phylogenetic analysis (*n* = 17)

**Table 4:** Sequencing results for *Mycoplasma* spp., using HBT-F and HBT-R primers, and their closest relationship, according to GenBank BLAST results.

Number of samples	Country	Reference haplotype	Reference host	Reference country	Reference accession number	Identity (in %)
18	A, F, R, S	*Candidatus* Mycoplasma haematomelis	*Meles meles anakuma*	Japan	AB848713	99.3–99.7
1	R	*Candidatus* Mycoplasma haematominutum	*Felis catus*	Italy	KR905451	100.0
5	C, G, R	*Mycoplasma* sp.	*Canis lupus familiaris*	Cambodia	ON620261	99.1–99.8
11	F, H, R	*Mycoplasma* sp.	*Procyon lotor*	USA	KF743729	96.0–96.2
50	B, C, F, G, H, I, R, S	*Mycoplasma* sp.	*Procyon lotor*	USA	KF743733	98.9–99.8
1	R	*Mycoplasma* sp.	*Felis silvestris silvestris*	Bosnia and Herzegovina	MF614158	100.0
8	G, H, R, S	*Mycoplasma* sp.	*Felis silvestris silvestris*	Bosnia and Herzegovina	MF614159	99.6–99.8

*A* Austria, *B* Bosnia and Herzegovina, *C* Croatia, *F* France, *G* Germany, *H* Hungary, *I* Italy, *R* Romania, *S* Serbia

Of the samples tested, 13 sequences of minor quality were excluded from further analysis (*n* = 94)

## Discussion

This study included a large (*N* = 220) number of examined badgers, originating from nine different countries in Southern, Western and Eastern Europe [58], thereby providing a representative cross-section of the badger population in continental Europe and its distribution [59]. It should be noted, however, that the sample sizes varied across the countries, ranging from two to 80 examined samples (Fig. 1).

The composition of pathogens identified in the samples examined in the present study is roughly comparable to that reported in other European studies. The prevalence of *T. pestanai* was high (56%) in our study compared to that reported in earlier studies where it ranged from 10% to 35% [28, 32, 60], but the majority of previous publications reporting *T. pestanai* originated from the UK [28, 32, 60]. Apart from these studies, in Europe, *T. pestanai* has only been found in two badgers from Italy, which already showed clinical symptoms prior to dying [29]. The recent detection of badger-associated *T. pestanai* in a dog from Germany suggests that the pathogen can be transmitted to other carnivores [31]. Therefore, the high prevalence of this parasite in the present study could represent a potential risk to both wild and domestic carnivores, especially when their immune status is compromised [29]. Further research is needed in this area to enable more precise conclusions.

In 2009, a new piroplasmid species was detected in a badger in Spain (GenBank accession number: FJ225390), which was considered to belong to *Babesia microti*-like organisms [21] and which was later found in several other studies and referred to as *Babesia* sp. badger type A by some authors [1, 16–19, 22, 25]. A different badger-associated *Babesia* species was designated as type B [1, 17, 22, 25]. In 2021, another *Babesia* species was found in one badger from the Netherlands [25] which was > 99% identical to a species found in a badger in an unpublished Chinese study (GenBank accession number: MG799847). These authors did not upload it to the GenBank but referred to it as *Babesia* sp. badger type C [25]. In the present study, all three badger-associated *Babesia* species were detected, with type A being the most prevalent (61%), followed by type C (43%) and type B (25%). Interestingly, *Babesia* sp. type C was not found in Austria, France and Germany, but only in Italy and the countries of Eastern Europe (Fig. 2). This geographical feature may be attributed to the natural barrier of the Alps, which separates north-western and south-eastern European countries, impacting wildlife and vectors. The unexpectedly high prevalence of type C, previously reported only in two other badgers from southern Italy and China [16], may be attributed to the large sample size and diverse geographical origin of the badgers examined, thus allowing for a more sensitive and differentiated analysis. Moreover, previous studies rarely examined samples from badgers south of the Alpine and Apennine belts, which could also explain the very low number of *Babesia* sp. type C found in past studies. Ixodid ticks are described as the primary vectors for *Babesia* spp. in Europe, and some of these vectors are prevalent in Alpine regions [61, 62]. While *Babesia* sp. showing 100% identity to type C was found in *Ixodes canisuga*, the fox tick, in Germany (GenBank accession number: JX679177), *Babesia* sp. badger types A and B have not yet been identified in ectoparasite vectors [1, 16–19, 22, 25]. However, the geographical distribution of type C could indicate, that this genotype needs a different vector than types A and B. More studies are needed to assess the vectors, definite hosts and the precise life cycle of this pathogen. Badger-associated *Babesia* spp. have also been detected in wolves (*Canis lupus*) [16], wildcats [26] and even dogs [19] in Europe. This indicates that the pathogens are not strictly specific to badgers and could therefore be a potential threat to both wild and domestic carnivores.

In contrast to other studies carried out in Europe, *A. phagocytophilum* was not detected in the present study [1, 40]. Three sequences were 99.5–100% identical to *Ehrlichia* sp. found in a dog from Hungary (GenBank accession number: MH020203) [41].

To the authors’ knowledge, only two previous studies reported hemoplasmas in badgers, with one describing hemoplasmas in a Japanese badger [38] and the other describing hemoplasmas in Eurasian badgers from Spain [37]. The high prevalence in the present survey is in concordance with the results reported from a study in Spain [36], where the prevalence of hemoplasmas was 57%. Similarly, a high diversity of *Mycoplasma* lineages was observed in the present study (Table 4).

To obtain more detailed genetic information, we selected 19 *Mycoplasma*-positive samples for performing an additional PCR in order to obtain a longer fragment of the* 16S* rRNA gene. The aim was to draw conclusions from the representative samples and to adapt the results of the remaining 88 sequences accordingly. However, the results differed significantly from one another, and DNA fragments from samples with 100% identity showed high deviations from results obtained with the primary PCR. Therefore, it was not possible to infer the *Mycoplasma* species of the samples from the first PCR based on the results of the representative 19 samples. The difference in results indicates the presence of co-infections with two or more *Mycoplasma* species in some of the badgers examined.

The prevalence of hemoplasmas was highest in Hungary, followed by Austria and Germany, and it was lowest in Italy. Due to the sparse body of literature available on hemoplasmas in badgers, a detailed comparison was not possible. The prevalence of hemoplasmas in European wild carnivores showed variable results, ranging from 2% in foxes (*Vulpes vulpes*) to 57% in badgers from Spain [26, 37, 63–65]. Although studies in domestic cats and dogs revealed a higher prevalence of hemoplasmas in warmer regions [46, 66], this difference does not appear as pronounced among wild carnivores. In contrast, variations in prevalence are more noticeable across different species. In our study, we observed a higher prevalence in colder regions compared to Mediterranean countries (e.g. Italy) (Fig. 3). This finding indicates that climatic conditions may not be as crucial in the transmission of hemoplasmas among wild carnivores as in pets. One possible explanation based on by new evidence is that hemoplasmas may not always require ectoparasite vectors, but may be transmitted by different mechanisms, such as fighting or social contact [67].

*Leishmania infantum* was previously identified in badgers from Italy and Spain with a prevalence ranging from 1.7% to 53% [1, 24, 68–71]. In the present study, however, despite examining large sample sizes from Mediterranean countries where *L. infantum* is endemic [72], none of the samples tested positive for this pathogen. Further studies on the prevalence of this parasite in the Eurasian badger population in Europe, especially in the Mediterranean region, could be of interest in the future due to the badger’s risk of being potential reservoir hosts of leishmaniasis [68].

Although we examined samples from countries where Filarioidea, such as *D. immitis* and *D. repens*, are described as endemic (e.g. Italy, Serbia, Hungary and Romania) [73–78], we did not detect any filarioid nematodes in our studies. Both *D. immitits* and *D. repens* have been recently detected in badgers [11, 12, 33, 34], which indicates that badgers are suitable hosts for *Dirofilaria* spp. It should be considered that the prevalence of *D. repens* in the two studies was quite low (10.6% in Russia and 1.9% in Poland), and to date, the presence of *D. immitis* in badgers has only been reported from Romania and Greece [12, 33].

*Rickettsia* spp. and *Bartonella* spp. were not detected in this study. Notably, *Rickettsia* spp. are not regularly tested for in badgers, and previous studies have primarily detected these pathogens in skin biopsies rather than spleen samples [20, 43, 79]. Regarding *Bartonella* spp., apart from one study from Spain that reported a prevalence of 12% [42], no other studies detected *Bartonella* spp. in badgers, including those focusing on vectors collected from badgers [20, 23, 80, 81]. Nevertheless, it should be noted that the sample sizes used in the latter four studies were small, ranging from three to 18 individuals. Due to the infestation of the badgers with multiple ectoparasites in the above-mentioned study from Spain [42], a determination of vectors for *Bartonella* sp. found in badgers is needed.

As wildlife-borne pathogens are responsible for > 70% of emerging zoonotic infectious diseases [82] and also play a crucial role as reservoirs for pathogens that can be transmitted to domestic animals [20], studies on VBPs in wildlife are becoming increasingly relevant. The badger-associated *Babesia* spp., *T. pestanai* and *Ehrlichia* sp. detected in this study are described as badger-specific pathogens. However, badger-associated *Babesia* spp. and *Ehrlichia* sp. as well as *T. pestanai* have been previously found in dogs [19, 31, 41], therefore posing a potential risk to pet dogs living in the vicinity of badgers. Hemoplasmas can infect a variety of mammals. The mycoplasmas identified in this study were similar to those of species detected in other wild and domestic carnivores. Since hemoplasmas have also been described in humans [83–85], further investigation of the pathogens in wild carnivores would be of interest.

Notably, the overall prevalence of pathogens was comparatively low in France and, in particular, in Italy (Fig. 3), although no significant correlation was observed between pathogen occurrence and the country of origin of the badgers. Given that many southern European countries are known for various endemic VBPs and their vectors [86, 87], it was anticipated that the prevalence of pathogens in these regions would be higher than that in more northern parts of Europe. However, it is important to note that much of the existing literature on VBPs in these countries has primarily focused on filarioid nematodes and *L. infantum*, both of which were not identified here [86, 87]. Regarding the low prevalence in Italy, it should be mentioned that the majority of individuals tested in Italy came from the southern region of Campania (*n* = 10), while the rest of the samples (*n* = 6) originated from the northern regions Veneto and Friuli-Venezia Giulia (Fig. 1). Although only four (40.0%) of these 10 badgers from southern Italy tested positive, all six (100.0%) badgers from the northern region tested positive for at least one pathogen. As VBPs and their vectors are quite widespread in southern Italian regions [88–90], this geographical distribution is rather surprising.

No statistically significant correlation was found between the presence of pathogens and the sex or age of the animals. Previous research examining the relationship between the presence of VBPs and factors such as sex or age has yielded contradictory results and shown considerable variability depending on the specific pathogen under investigation [91–96].

## Conclusions

The large sample size and diverse study populations in this research provide valuable insights into the distribution and epidemiology of the pathogens analyzed in Eurasian badgers. Several VBPs identified in the present study are highly similar to those found in domestic animals, such as dogs, which suggests that badgers may pose a threat to other wildlife and domestic animals in their vicinity as potential reservoirs of these pathogens. Continued surveillance is essential to monitor VBPs in wildlife and to assess their impact on other wildlife, domestic animals and human health.

## Supplementary Information

Additional File 1: Table S1. PCR protocols and cycling conditions used in the present study. Table S2. Positivity to one or more pathogens in badgers in continental Europe in relation to sex, age, country of origin, year of sample collection, and month of sample collection.

Additional File 2: Figure S1. Bayesian Inference tree based on 16S rRNA (963 nucleotide positions) sequences of selected Mycoplasma spp. Bayesian posterior probabilities and Maximum Likelihood bootstrap values are provided for most nodes. Accession numbers, species name, host, and country are provided for every sequence if available. Sequences that are written in bold are from Eurasian badgers and sequences marked in red were obtained in this study. The scale bar indicates the expected mean number of substitutions per site according to the model of sequence evolution applied.

## Data Availability

The data presented in this study are contained within the article and supplementary material. Additional data can be provided on request.

## Abbreviations

BI: Bayesian inference
BIC: Bayesian inference criterion
ML: Maximum likelihood
RFLP: Restriction fragment length polymorphism
VBP: Vector-borne pathogen
