# Supplementary material for: Molecular analysis of vector-borne pathogens in Eurasian badgers (Meles meles) from continental Europe
Source: Parasit Vectors. 2024 Nov 4;17:451. doi: 10.1186/s13071-024-06515-y (PMC11536541; doi:10.1186/s13071-024-06515-y)
Supplement: Supplementary file 1 — Additional File 1: Table S1. PCR protocols and cycling conditions used in the present study. Table S2. Positivity to one or more pathogens in badgers in continental Europe in relation to sex, age, country of origin, year of sample collection, and month of sample collection. [file 13071_2024_6515_MOESM1_ESM.docx]

**Additional File 1: Table S1.**  PCR protocols and cycling conditions used in the present study.

| **Target organism (genetic marker)** | **Mastermix protocol** | | | **Cycling Conditions** | | | |
| --- | --- | --- | --- | --- | --- | --- | --- |
|  | **Reagents** | **Unit Reagent** | **Quantity (µl) per tube** | **Temperature (°C)** | **Time** | **Step** | |
| ***Mycoplasma* spp. (16S rRNA)** |  |  |  |  |  |  |  |
|  | H_2_O |  | 12.675 | 94 | 2’ | Initial denaturation |  |
|  | green reaction buffer | 5 x | 5 | 95 | 1' | 40 x |  |
|  | dNTPs | 25 mM | 0.2 | 60 | 1’ |  |  |
|  | TagPolymerase (GoTaq) | 5 u/µl | 0.125 | 72 | 1’ |  |  |
|  | Primer Forward | 10 pmol/µl | 1 | 72 | 7’ | Final extension |  |
|  | Primer Reverse | 10 pmol/µl | 1 | 15 | ~ | Storage |  |
|  | Template | 5 µl | 5 |  |  |  |  |
| **Piroplasmida**  **(18S rRNA)** |  |  |  | **Nest 1** |  |  |  |
|  | H_2_O |  | 30.35 | 94 | 2’ | Initial denaturation |  |
|  | green reaction buffer | 5 x | 10 | 94 | 45’’ | 40 x |  |
|  | dNTPs | 25 mM | 0.4 | 52 | 45’’ |  |  |
|  | TagPolymerase (GoTaq) | 5 u/µl | 0.25 | 72 | 1’ |  |  |
|  | Primer Forward | 100 pmol/µl | 2 | 72 | 5’ | Final extension |  |
|  | Primer Reverse | 100 pmol/µl | 2 | 15 | ~ | Storage |  |
|  | Template | 5 µl | 5 | **Nest 2** |  |  |  |
|  |  |  |  | 94 | 2’ | Initial denaturation |  |
|  |  |  |  | 94 | 45’’ | 35 x |  |
|  |  |  |  | 62 | 45’’ |  |  |
|  |  |  |  | 72 | 1’ |  |  |
|  |  |  |  | 72 | 5’ | Final extension |  |
|  |  |  |  | 15 | ~ | Storage |  |

**Additional File 1: Table S1.**  PCR protocols and cycling conditions used in the present study (cont.).

| **Target organism (genetic marker)** | **Mastermix protocol** | | | **Cycling Conditions** | | | |
| --- | --- | --- | --- | --- | --- | --- | --- |
|  | **Reagents** | **Unit Reagent** | **Quantity (µl) per tube** | **Temperature (°C)** | **Time** | **Step** | |
| **Trypanosomatida (18S rRNA)** |  |  |  | Nest 1 + 2 |  |  |  |
|  | H_2_O |  | 14.675 | 94 | 5’ | Initial denaturation |  |
|  | green reaction buffer | 5 x | 5 | 94 | 1’ | 35 x |  |
|  | dNTPs | 25 mM | 0.2 | 56 | 1’ |  |  |
|  | TagPolymerase (GoTaq) | 5 u/µl | 0.125 | 72 | 1’ |  |  |
|  | Primer Forward | 10 pmol/µl | 2 | 72 | 5’ | Final extension |  |
|  | Primer Reverse | 10 pmol/µl | 2 | 15 | ~ | Storage |  |
|  | Template | 5 µl | 1 |  |  |  |  |
| ***Bartonella* spp. (16S-23S rRNA)** |  |  |  |  |  |  |  |
|  | H_2_O |  | 12.675 | 95 | 10’ | Initial denaturation |  |
|  | green reaction buffer | 5 x | 5 | 95 | 15'’ | 30 x |  |
|  | dNTPs | 25 mM | 0.2 | 60 | 1’ |  |  |
|  | TagPolymerase (GoTaq) | 5 u/µl | 0.125 | 72 | 20’’ |  |  |
|  | Primer Forward | 20 pmol/µl | 1 | 72 | 5’ | Final extension |  |
|  | Primer Reverse | 20 pmol/µl | 1 | 15 | ~ | Storage |  |
|  | Template | 5 µl | 5 |  |  |  |  |
| ***Rickettsia* spp. (23S-5S rRNA)** |  |  |  |  |  |  |  |
|  | H_2_O |  | 12.675 | 96 | 4’ | Initial denaturation |  |
|  | green reaction buffer | 5 x | 5 | 94 | 1’ | 35 x |  |
|  | dNTPs | 25 mM | 0.2 | 52 | 1’ |  |  |
|  | TagPolymerase (GoTaq) | 5 u/µl | 0.125 | 72 | 2’ |  |  |
|  | Primer Forward | 10 pmol/µl | 1 | 72 | 3’ | Final extension |  |

**Additional File 1: Table S1.**  PCR protocols and cycling conditions used in the present study (cont.).

| **Target organism (genetic marker)** | **Mastermix protocol** | | | **Cycling Conditions** | | | |
| --- | --- | --- | --- | --- | --- | --- | --- |
|  | **Reagents** | **Unit Reagent** | **Quantity (µl) per tube** | **Temperature (°C)** | **Time** | **Step** | |
| ***Rickettsia* spp. (23S-5S rRNA)** |  |  |  |  |  |  |  |
|  | Primer Reverse | 10 pmol/µl | 1 | 15 | ~ | Storage |  |
|  | Template | 5 µl | 5 |  |  |  |  |
| **Anaplasmataceae (16S rRNA)** |  |  |  |  |  |  |  |
|  | H_2_O |  | 11.175 | 95 | 2’ | Initial denaturation |  |
|  | green reaction buffer | 5 x | 5 | 94 | 1’ | 30 x |  |
|  | dNTPs | 25 mM | 0.2 | 54 | 3’’ |  |  |
|  | MgCl_2_ | 25 mM | 1.5 | 72 | 30’’ |  |  |
|  | TagPolymerase (GoTaq) | 5 u/µl | 0.125 | 72 | 5’ | Final extension |  |
|  | Primer Forward | 10 pmol/µl | 1 | 15 | ~ | Storage |  |
|  | Primer Reverse | 10 pmol/µl | 1 |  |  |  |  |
|  | Template | 5 µl | 5 |  |  |  |  |
| **Filarioidea**  **(COI)** |  |  |  |  |  |  |  |
|  | H_2_O |  | 14.175 | 95 | 2’ | Initial denaturation |  |
|  | green reaction buffer | 5 x | 5 | 95 | 45'’ | 35 x |  |
|  | dNTPs | 25 mM | 0.2 | 52.3 | 45’’ |  |  |
|  | TagPolymerase (GoTaq) | 5 u/µl | 0.125 | 72 | 1’ |  |  |
|  | Primer Forward | 100 pmol/µl | 0.25 | 72 | 5’ | Final extension |  |
|  | Primer Reverse | 100 pmol/µl | 0.25 | 15 | ~ | Storage |  |
|  | Template | 5 µl | 5 |  |  |  |  |

**Additional File 1: Table S2.**  Positivity to one or more pathogens in badgers in continental Europe, in relation to sex, age, country of origin, year of sample collection, and month of sample collection.

| **Variables** | **Negative** | **Positive** | **Proportion in %** | **95% CI** | **Statistical analysis** | |
| --- | --- | --- | --- | --- | --- | --- |
| **Sex**^a^ |  |  |  |  |  |  |
| Male | 10 | 100 | 90.91 | 0.84 – 0.95 | χ2 = 72.01, *df* = 1, *P* < 0.001* |  |
| Female | 6 | 63 | 91.30 | 0.81 – 0.96 | χ2 = 45.45, *df* = 1, *P* < 0.001* |  |
| **Age**^b^ |  |  |  |  |  |  |
| Adult | 14 | 119 | 89.47 | 0.83 – 0.94 | χ2 = 81.32, *df* = 1, *P* < 0.001* |  |
| Juvenile | 0 | 21 | 100 | 0.81 – 1 | χ2 = 19.05, *df* = 1, *P* < 0.001* |  |
| **Origin**^c^ |  |  |  |  |  |  |
| Austria | 7 | 7 | 100 | 0.93 – Inf^f^ | *P* = 0.047*, OR = Inf^f^ |  |
| Bosnia and Herzegovina | 2 | 2 | 100 | 0.08 – Inf^f^ | *P* = 0.467, OR = Inf^f^ |  |
| Croatia | 22 | 22 | 100 | 4.58 – Inf^f^ | *P* < 0.001*, OR = Inf^f^ |  |
| France | 37 | 44 | 84.09 | 2.02 – 15.41 | *P* < 0.001*, OR = 5.22 |  |
| Germany | 16 | 16 | 100 | 3.06 – Inf^f^ | *P* < 0.001*, OR = Inf^f^ |  |
| Hungary | 7 | 7 | 100 | 0.93 – Inf^f^ | *P* = 0.047*, OR = Inf^f^ |  |
| Italy | 10 | 16 | 62.50 | 0.42 – 6.96 | *P* = 0.542, OR = 1.65 |  |
| Romania | 75 | 80 | 93.75 | 5.66 – 49.56 | *P* < 0.001*, OR = 14.86 |  |
| Serbia | 24 | 26 | 92.31 | 2.48 – 112.06 | *P* < 0.001*, OR = 11.66 |  |
| **Year**^d^ |  |  |  |  |  |  |
| 2010 | 1 | 1 | 100 | 0.01 – Inf^f^ | *P* = 1, OR = Inf^f^ |  |
| 2011 | 1 | 1 | 100 | 0.01 – Inf^f^ | *P* = 1, OR = Inf^f^ |  |
| 2015 | 1 | 1 | 100 | 0.01 – Inf^f^ | *P* = 1, OR = Inf^f^ |  |

^a^ The sex of 41/220 animals remained unknown

^b^ The age of 66/220 animals remained unknown

^c^ Country of origin

^d^ Year of sample collection, the year of sample collection remained unknown for 12/220 animals

^e^ Month of sample collection, the month of sample collection remained unknown for 33/220 animals

^f^ Limitation in data due to small sample size

* Statistically significant

**Additional File 1: Table S2.**  Positivity to one or more pathogens in badgers in continental Europe, in relation to sex, age, country of origin, year of sample collection, and month of sample collection (cont.).

| **Variables** | **Negative** | **Positive** | **Proportion in %** | **95% CI** | **Statistical analysis** |
| --- | --- | --- | --- | --- | --- |
| **Year**^d^ |  |  |  |  |  |
| 2016 | 12 | 12 | 100 | 2.07 – Inf^f^ | *P* = 0.003*, OR = Inf^f^ |
| 2017 | 15 | 15 | 100 | 2.80 – Inf^f^ | *P* < 0.001*, OR = Inf^f^ |
| 2018 | 34 | 33 | 97.06 | 4.81 – 1371.33 | *P* < 0.001*, OR = 32.16 |
| 2019 | 23 | 19 | 82.61 | 1.27 – 21.73 | *P* = 0.010*, OR = 4.65 |
| 2020 | 112 | 101 | 90.18 | 4.57 – 19.91 | *P* < 0.001*, OR = 9.13 |
| 2021 | 9 | 8 | 88.89 | 0.73 – 392.66 | *P* = 0.091*, OR = 7.45 |
| **Month**^e^ |  |  |  |  |  |
| Jan | 7 | 7 | 100 | 0.93 – Inf^f^ | *P* = 0.047*, OR = Inf^f^ |
| Feb | 16 | 17 | 94.12 | 1.95 – 706.86 | *P* = 0.002*, OR = 15.28 |
| Mar | 10 | 12 | 83.33 | 0.77 – 54.11 | *P* = 0.076, OR = 4.79 |
| Apr | 9 | 9 | 100 | 1.37 – Inf^f^ | *P* = 0.012*, OR = Inf^f^ |
| May | 20 | 21 | 95.24 | 2.60 – 863.61 | *P* < 0.001*, OR = 19.24 |
| Jun | 29 | 33 | 87.88 | 2.16 – 30.96 | *P* < 0.001*, OR = 7.11 |
| Jul | 29 | 32 | 90.63 | 2.54 – 53.35 | *P* < 0.001*, OR = 9.46 |
| Aug | 14 | 15 | 93.33 | 1.63 – 628.39 | *P* = 0.007*, OR = 13.31 |
| Sep | 11 | 11 | 100 | 1.84 – Inf^f^ | *P* = 0.005*, OR = Inf^f^ |
| Oct | 14 | 15 | 93.33 | 1.63 – 628.39 | *P* = 0.007*, OR = 13.31 |
| Nov | 7 | 9 | 77.78 | 0.46 – 41.66 | *P* = 0.231, OR = 3.34 |
| Dec | 5 | 6 | 83.33 | 0.35 – 274.62 | *P* = 0.316, OR = 4.59 |

^a^ The sex of 41/220 animals remained unknown

^b^ The age of 66/220 animals remained unknown

^c^ Country of origin

^d^ Year of sample collection, the year of sample collection remained unknown for 12/220 animals

^e^ Month of sample collection, the month of sample collection remained unknown for 33/220 animals

^f^ Limitation in data due to small sample size

* Statistically significant
